# Dual flatness filter design for single energy photon beam of medical linear accelerator – Rationale and efficacy

**DOI:** 10.1002/acm2.70349

**Published:** 2025-11-18

**Authors:** Ramamoorthy Ravichandran, Tarani Mondal, Tamilarasan Mani, Manikandan Palanisamy, G. V. Subrahmanyam

**Affiliations:** ^1^ Medical Physics Unit Department of Radiation Oncology Cachar Cancer Hospital and Research Centre Silchar Assam India; ^2^ Panacea Medical Technologies Ltd. Malur Karnataka India

**Keywords:** dual flatness filters, filter transmission, low energy linac, Siddharth II

## Abstract

**Background:**

Linear accelerators used for cancer treatments, in general, have one flattening filter (FF) for each x‐ray energy. In a newly developed 6 MV low energy linac model, beam optimization at higher dose rates is achieved by two FFs.

**Purpose:**

To achieve optimization in higher dose rates for smaller fields with FF, one small flattening filter (SF) for fields covering up to 16 × 16 cm^2^ and another large flattening filter (LF), to provide flattened fields till 30 × 30 cm^2^ are made available. A smaller thickness filter selectively flattens central part of the beam.

**Methods:**

Recently, a new generation low energy Siddharth II model 6 MV linac is manufactured by M/s Panacea Medical Technologies Pvt Ltd in India, with two FFs. Experimentally measured dose rates with SF and FF vis‐à‐vis un‐flattened open beam were compared with theoretical estimates. Furthermore, a comparison is made with beam characteristics of a True Beam Varian linear accelerator for 6 MV beam.

**Results:**

As beam hardening is expected with FF, it was taken appropriate to consider 1.20 MeV mean energy for 6 MV bremsstrahlung continuous spectrum, before entry to FF. Measured transmissions of 0.8140 and 0.4470 for SF and LF, respectively, compare well with theoretically estimated 0.7904 and 0.4130. A dose rate enhancement factor 1.821 is achieved along with better flatness for smaller fields. The measured factor 0.4470 for large filter for Siddharth II, is similar to a transmission of 0.5100 for True Beam 6 MV photons.

**Conclusion:**

As the first of its kind, the thin FF covers smaller solid angle of the total beam, giving higher MU/min in small fields, which might help for radical treatments. Also, there will be less power input requirements because of gain in dose rate.

## 1 INTRODUCTION

In the 1980s, linac manufacturers introduced machines with different types of electron beam transport to strike the targets, to get ‘bremsstrahlung’ photon beams. In these designs, alignment of the central line of the flattening filter (FF) became crucial. At the start of this millennium, the addition of multi‐leaf collimators (MLC) enabled the development of “photon beam‐lets” to achieve “small” fields, either by static multiple fields or by dynamic moving slit beams, which could not be achieved by jaws alone. Data acquisition by radiation field analysers (RFA) and high speed treatment planning computers (TPS), made intensity modulation (IM) and volume modulation (VM) of radiation dose possible in 3‐Dimensional (3D) irregular volumes.

While optimizing dose in very small volumes, such as in “radiosurgery” (single high dose treatments), precision and rapid dose delivery led to introduction of “filter‐free” photon beams with a central hump. Irregular dose distributions could be handled by TPS to achieve homogeneity. This was possible because TPS could calculate volume doses from the individual beam‐lets, achieving homogeneity in “planned small tumor volumes (PSTV)”. The present‐day technology is to manufacture digital linacs in which pulsed radiation beam management could be achieved, to modulate beam in time space. In this background, it could be inferred that removal of flatness filter (filter‐free beam) gives high intensity beam at the cost of flatness. Introduction of the FF make the beam flat by altering intensity as well as hardening the beam.

Most of the manufactured high energy linac photon beams use a single FF, which have central conical part of FF aligned to the arrow head dose profile, and machined based on the acquired radiation profile at a fixed depth in a water phantom. Such single FF give flatness for fields from minimum field size of 4 cm × 4 cm to maximum field size of 40 cm × 40 cm at isocenter (at 1 m). Accepted flatness is measured using difference between maximum and minimum dose points on the beam profile, within the central 80% of beam width. The principle of fabrication of FF was explained well^1^ in literature. Flatness is expressed by finding maximum in terms of accepted “± variations” of intensity in beam profiles for many field sizes, and it is customary to accept a specification of ± 3% flatness and ± 2% for symmetry in a reference field^2^ of 20 cm × 20 cm at 10 cm reference depth in water at 100 cm focus to reference depth distance. A new version 6 MV linear accelerator Siddharth II introduced in clinical radiotherapy in the last 3 years utilizes two beam FFs.^3^ So far, nine machines are in clinical use, and five more are under installation. A comparison of dose rates achieved with this design and their rationale is presented.

## 2 MATERIALS AND METHODS

### 2.1 Mean photon energy in 6 MV linacs (theoretical background)

The polar diagram^4^ of intensity distribution has an arrowhead shape, with spreads up to a certain solid angle, with both energy and intensity spreads. A forward hump towards the central axis is made flat by a high density filter located just below the primary collimator aperture. FF also has an effect on differential intensity reduction, as we move away from central beam line towards the edge of the beam. Edges of the beam mostly have lower energy, and less thickness of filter material in the edges leave “horns” which will get suppressed with depth of the medium, due to radial line effects.

It was reported earlier^5^ that mean photon energy is lower than 1/3^rd^ of maximum energy of high speed accelerated electrons (<2 MeV for 6 MV beam). The energy spectrum of low energy 6 MV linacs was investigated by many other authors.^6^, ^7^, ^8^, ^9^ Extracts of their findings are summarized in Table 1. With the above background, an attempt was made to derive higher dose rate by beam management with two flattening filters (dual FFs) in 6 MV photon linear accelerator. One FF makes use of smaller central thickness for smaller fields, and larger central thickness FF for larger fields.

**TABLE 1:** Composition of 6 MV linac x‐ray beam.

No.	Method	6 MV Linac Photons Spectral distribution	Remarks
1	By using copper absorbers, energy response function and Laplace transform analysis.^6^	1.7 MeV ‐100%, 2.5 MeV ‐70%, 3.0 MeV ‐ 50%, 3.6 MeV ‐30% 4.0 MeV ‐10% and no detectable energy > 4.6 MeV.	Mean *µ*_en_/ρ _Copper =_ 0.04443 cm^2^/g **(1.7 MeV)**. *µ*_en_/ρ_Copper_ for **6 MeV** mono‐energetic beam = 0.0318 cm^2^/g.
2	Monte Carlo computational results yielded following results^7^	1.67 MeV (at *d* = 0), 1.86 MeV (*d* = 20 cm). **3** × **3 cm^2^ field**. 1.47 MeV(at *d* = 0), 1.27 MeV (*d* = 20 cm). **10** × **10 cm^2^ field**. 1.21 MeV(at *d* = 0), 0.694 MeV (*d* = 20 cm). **40** × **40 cm^2^ field**.	Clinac 2300, 6 MV beam, copper target, copper FF, tungsten primary collimator, and jaws. Results extendable to M/s Varian linac models Clinac 2100, 1800, 21 Ex, 23 Ex.
3	Spectroscopy with High Purity Germanium Scintillator and Monte Carlo method.^8^	0.8 MeV 100%), 1.0 MeV (90%) 1.4 MeV (50%) and > 1.8 MeV, the intensity drops < 30% to 5% at 5 MeV.	Clinac 21Ex model, M/s Varian linac.
4	Small field 6 MV x‐ray spectrum was evaluated with objective to investigate second cancer risk adjacent to radiotherapy radiotherapy‐treated volume.^9^	1.20MeV mean energy of x‐ray photons for small fields, in the central axis at *d* = 1.5 mm.	Did not mention information on filtration. The clinical significance of small fields is not highlighted. 1–10 KeV secondary electrons liberated by photons similar to cobalt‐60 radiation^9^

### 2.2 Design and specifications of linear accelerator

A new design, Siddharth II model 6 MV Linear Accelerator is manufactured by M/s Panacea Medical Systems Ltd (PMT), Malur, Kolar District, Karnataka. These machines received approval and licensed by Atomic Energy Regulatory Board (AERB), the Regulatory Authority (RA) in India for clinical use of linacs, under safety code.^10^, ^11^, ^12^ This model is International Electro‐technical Commission (IEC) compliant.^3^ The machine head is mounted on a ring type gantry to achieve highest isocentric accuracy, and rotated with a computer controlled geared motor and in‐built Radiation Field Analyser (RFA) with the gantry assembly. This linac is operated with 400 mA cathode emission current, and peak Radiofrequency (RF) output power of 2.6 MW.

### 2.3 Design of 2 photon flatness filters

**FIGURE 1:**
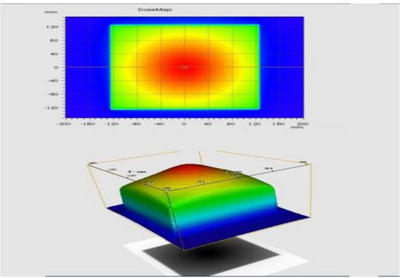
Colour Wash of beam from the x‐ray target before flattening (displayed in treatment planning system). The radiation profiles need central part attenuation to achieve perfect flat isodose pattern, in inline, crossline, and diagonal directions. The metallic flattening filter, therefore, has radial differential attenuation of excess intensity.

**FIGURE 2:**
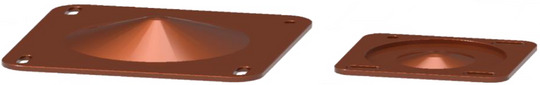
LF and SF in Copper. LF, large size flattening filter; SF, small size flattening filter.

Siddharth II has two FFs, smaller filter for intermediate field sizes, and a larger filter for remaining fields. In Figure 1, the practical radiation emission pattern arising from the transmission target is illustrated. To reduce the central hump and make the beam flat within specified limits, it is a general practice to have an overall flux control by a single large FF for all field sizes. Copper or Stainless Steel is used as FF, and different FFs will be designed for each photon energy in a multi‐energy linac. A new innovation is made recently in the design of Siddharth II linacs, by introducing one FF of smaller width, which can handle small fields (SF) in the centre, and another FF of larger width (LF) to cover full beam, so that SF might give advantageous situation to enhance the dose rates for smaller group of field sizes. Figure 2 shows the SF and LF made up of copper, which clearly shows that SF is thinner, with smaller dimensions.

**FIGURE 3:**
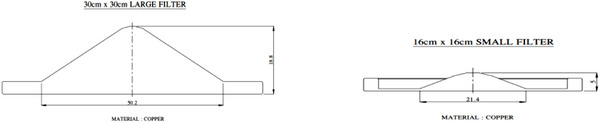
Section view of SF and LF. Base 50.2 mm, 18.8 mm thick (LF), Base 21.4 mm, 5 mm thick (SF). LF, large size flattening filter; SF, small size flattening filter.

During initial stages, when the linac was energized, measurements were carried out in radiation profiles. The increased intensity in the central part of the beam is corrected based on empirical percent of water transmission, rough Copper Equivalent thickness calculated, and filter inserted, and the profile measured again (Refer Table 2, right column for approximate transmission). The design was standardized after validating measured data, on completion of each equipment by the production department of PMT. Figure 3 shows the thickness of the small and large flattening filters (SF, LF), 0.5 and 1.88 cm, respectively, as per factory documents. Triangular SF and LF have base widths of 2.5 and 5 cm, and derived angles 26.5° and 49° with respect to the base.

**TABLE 2:** Theoretical estimates of transmission in SF and LF.

Attenuation calculations	Attenuated intensity estimates
**For Eav = 1.20 MeV** **I_SF_/ I_0_ = e^–0.0525^** ^×^ **^8.96^** ^×^ **^0.5^ (1)** **A) Transmission SF** = **0.7 904** **I_LF_/ I_0_ = e^–0.0525^** ^×^ **^8.96^** ^×^ **^0.5^ (2)** **Transmission LF** = **0.4 130** As beam hardening is expected with FF, it was taken appropriate to consider 1.20MeV mean energy for 6 MV bremsstrahlung continuous spectrum.	0.5 cm Cu; 4.48 cm equivalent water 1.88 cm Cu; 16.84 cm equivalent water For 6 MV intensity decrease in water equivalent media is 4% per cm.^2^ Therefore, a reduction of 67.4% open beam radiation intensity by LF and 17.9% open beam radiation intensity by SF is an approximation. A gain of 50% is theoretically achievable by the use of SF for less field dimension.
**For Eav = 1.70 MeV** **I_SF_/ I_0_ = e^–0.0444^** ^×^ **^8.96^** ^×^ **^0.5^ (1)** **B) Transmission SF** = **0.8200** **I_LF_/ I_0_ = e^–0.0525^** ^×^ **^8.96^** ^×^ **^0.5^ (2)** **Transmission LF** = **0.4741** *Case B is calculated for documentation, because if 1.70MeV is the bremsstrahlung energy at the target, there will be marginal increase in transmission through SF and LF*.	Small FF(SF) compared with Large FF(LF) (**Dose rate enhancement factor**) Excess transmission ratio I**_SF_**/I**_LF_** **Case A 0.7904/0.4130 = 1.9138** **(91% excess intensity)** **Case B 0.8200/0.4741 = 1.7296** **(73% excess intensity)**

**FIGURE 4:**
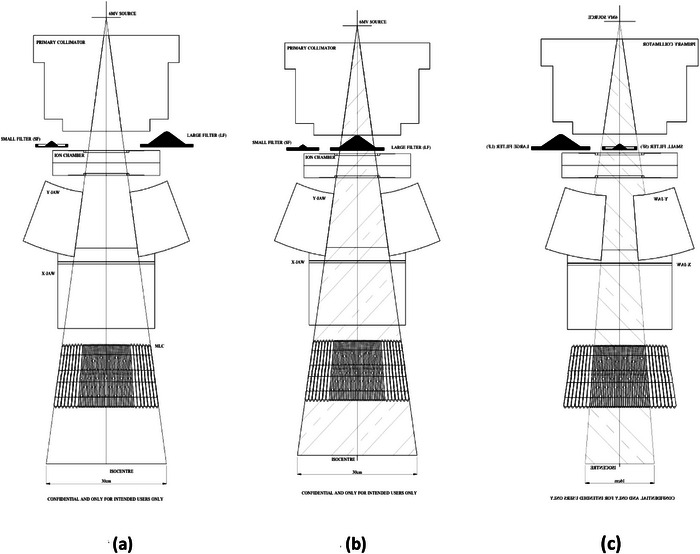
(a) Geometry of flattening filter free beam, with SF, and LF left and right of FFF condition. (b) Larger FF (LF) in position covering full beam for 30 × 30, full beam transmission in LF. (c) Smaller FF (SF) in position for fields upto 16 × 16. Only part of beam allowed through SF. LF, large size flattening filter; SF, small size flattening filter.

The geometry of flatness filters with respect to open FFF beam in the Siddharth II linac is shown in Figure 4a–c. SF and LF filters are on both left and right sides of the open portal (Figure 4a), Larger FF (LF) in position can give flatness for full beam for 30 × 30, covering more solid angle. Smaller FF (SF) in position can give flatness for Field Sizes up to 16 × 16. Beyond these small fields, an interlock will prevent use of SF, and LF will come into position for larger fields. For higher specification linacs, Siddharth II Superia model with 5 mm MLC resolution is designed at iso‐centre for fields upto 16 × 16 cm^2^, with 32 pairs of leaves, and remaining 14 pairs of leaves at 10 mm resolution (total 92 leaves). Therefore, a higher dose rate is needed with small flatness filter (SF).

### 2.4 Theoretical estimate of dose rate increase

The approximate dose rate increase is calculated theoretically based on the *µ*/*ρ*‐value estimates (Table 2). For the exit beam from the copper flattener, an earlier work^7^ estimated *µ*/ρ**_Cu_** = 0.0443 cm^2^/g for 1.70 MeV mean photon energy. With ρ**_Cu_ **= 8.96 g/cm^3^, the linear attenuation coefficient is *µ* = 0.3969 cm^−1^ at 1.70 MeV. Another Monte Carlo computations^8^ estimated an average photon energy 1.20 MeV at the surface of water medium at 1.5 mm depth. Referred value for 1.20 MeV [eqn.1] *µ*/ρ**_Cu_** = 0.0525 cm^2^/g and therefore *µ* = 0.4704 cm^−1^. Mean energy before FF is same as the energy reaching the surface of the medium without FF; Mean energy after FF is same as the energy at the surface of the medium with FF. For transmission calculations, therefore, *µ* = 0.4704 cm^−1^ at 1.20 MeV is used. The method to estimate Dose Rate Enhancement Factor (DREF) for SF vis‐à‐vis LF is based on exponential attenuation equations. I_0_ incident intensity on SF and LF as *I*_SF_ and *I*_LF_ are transmitted intensities, the DREF estimates are achieved (Table 2).

### 2.5 Measurement of flatness filter transmissions

To establish the efficacy of the small thickness flatness filter (SF) with regard to large thickness filter, experimental measurements of transmission were carried out in Siddharth II, for field sizes varying from 2 × 2 cm^2^ to 30 × 30 cm^2^. Measurements of radiation intensity were carried out, at 10 cm depth in water phantom, with Focus Chamber Distance (FCD) 100 cm using the built‐in RFA earlier described.^12^ A calibrated 0.04 cc ion chamber (Sun Nuclear, 538912 /01‐001 type), along with dual channel electrometer (Sun Nuclear 1014000‐OZ Type), was used. There was no leakage in the chamber, and necessary corrections were made on the meter reading to convert to absorbed dose. A pulse repetition frequency (PRF) 150 setting on the control console was kept during beam ON, for FFF, SF, and LF settings, which represents nominal 600 MU/min as per factory settings. Radiation exposures were repeated for all field sizes for 1 min. Integrated mode (MU) was not used, because the **ʃ** MU is expected to cumulate standard amount of charge for reproducible radiation quantity, and yield the same readings irrespective of the filter transmission. The transmitted dose for different fields and filter settings is tabulated. The ratio of measured doses under SF and LF is quantified against open beams (FFF exposures). The transmission of radiation under SF and LF compared to the effect of different FFs.

### 2.6 Estimates of beam profiles and filter attenuations in TrueBeam

The radiation beam profiles of TrueBeam Linac (Varian Model SVC) at our site in Silchar, for 6 MV, FFF beam, and FF beam are measured with RFA (Sun Nuclear) with cylindrical phantom and 0.125 cc ion chamber. From this the FF attenuation factors are evaluated. As these models use only one FF for 6 MV photon energy for minimum to maximum field sizes generated by collimator jaws (40 × 40 cm^2^), only one transmission factor is available.

## 3 RESULTS

### 3.1 Increase in dose rate (theoretical estimates)

The relative intensities as per specifications mentioned by the manufacturer along the central axis for reference field 10 × 10 are compared and shown in Table 3. The DREF could be seen as per catalogue, as 600 MU/min for SF and 300 MU/min for LF (a Factor of 2.000). This matches with theoretical approximation 1.914 (within 4.5%) as calculated by Equation (3) (Table 2, Case A). Transmission factor 0.7904, corresponds to 632 MU/min for 800 MU/min in a filter‐free setting, agrees with manufacturer's specification of 600 MU/min for fields upto 16 × 16. A transmission factor of 0.4130, corresponding to 330.4MU/min for 800 MU/min with a specification of 300 MU/min for fields upto 30 × 30 cm. A clear advantage is projected by a DREF factor 2, without compromising flatness because of better optimization in the built in design of SF in this linac. This will also help in lower power utilization related to the cathode and heating of the target, while selecting 600 MU/min.

**TABLE 3:** Specified dose rates available for clinical purposes in specifications.

Beam	D_max_ cm	%DD _10_ _cm_	FFF	SF for 16 × 16 cm^2^	LF for 30 × 30 cm^2^
**6**× **FFF**	**1.24**	**63.8%** (± 2%) 10 × 10 field	**50–800 MU/min** For 10 × 10 field	Not specified	Not specified
**6**× **FF (SF)** Upto 16 × 16	**1.34**	**64.5%** (± 2%) 10 × 10 field	Not specified	**30–600 MU/min** For 10 × 10 field	Not specified
**6**× **FF (LF)** (16 × 16 ‐30 × 30)	**1.50**	**67.5%** (± 2%) 10 × 10 field	Not specified	Not specified	**30–300 MU/min** for 10 × 10 field

***Note*: Dose rate enhancement factor as per specifications SF to LF till 16 cm** × **16 cm Field—Factor 2.000**.

### 3.2 Beam profiles for SF and LF

**FIGURE 5:**
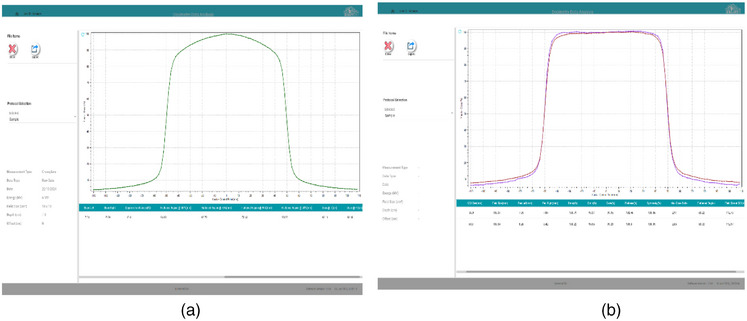
Measured flatness curves at 10 cm depth for 10 × 10 cm^2^ field size at depth 10 cm. (a) For open beam without flatness filter. (b) for SF beam (top curve), for LF beam (bottom curve). It is seen that SF flatness filter of 16 × 16 cm^2^ is able to provide better. Flatness than LF flatness filter of 30 × 30 cm^2^, providing flatness for small field of 10 × 10. LF, large size flattening filter; SF, small size flattening filter.

**FIGURE 6:**
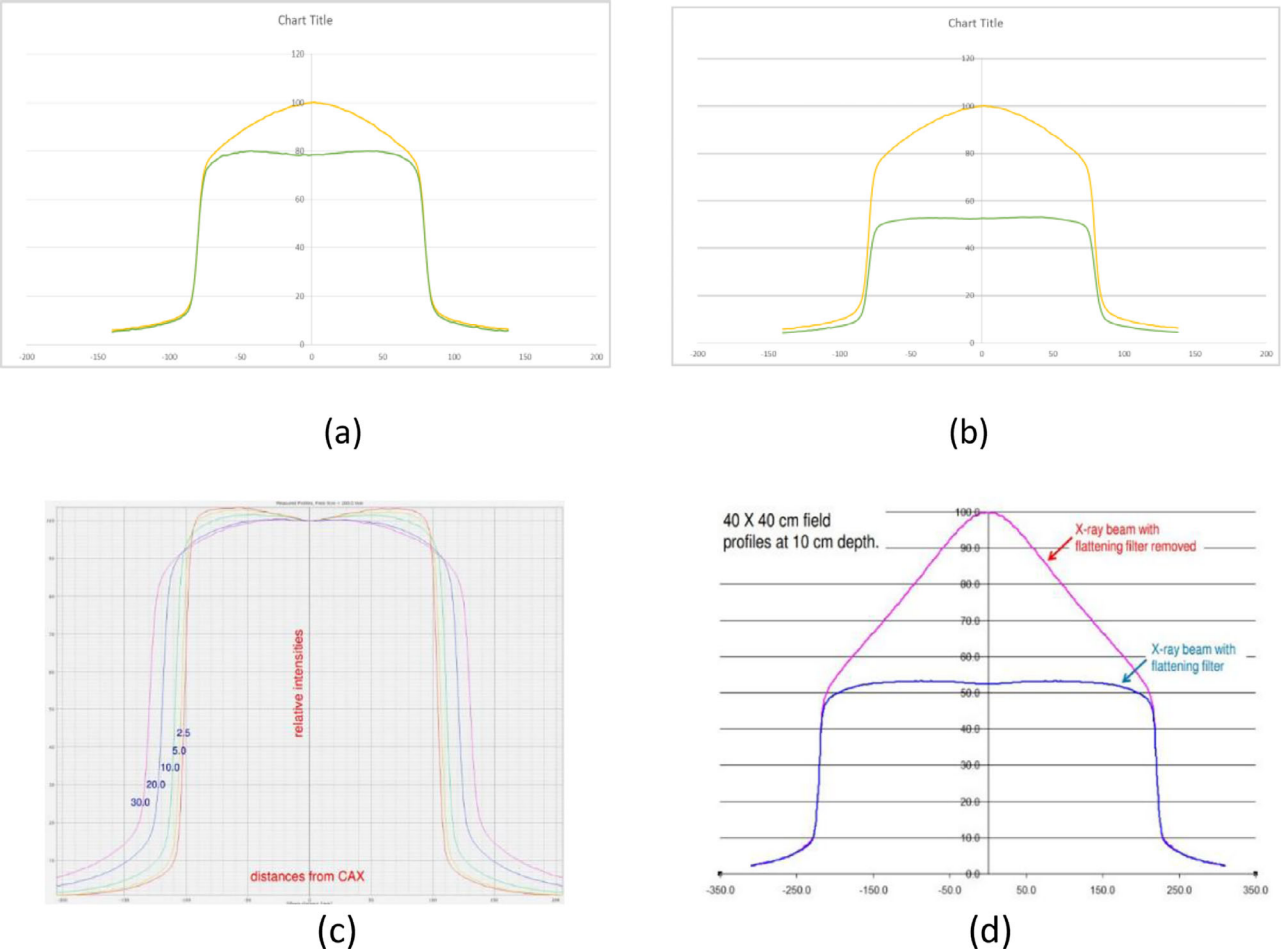
(a) Siddharth II measured radiation beam profiles for 6 MV FFF and FF for 16 × 16 cm^2^. (b) Siddharth II Measured Radiation Beam Profiles for 6 MV FFF and FF for 30 × 30 cm^2^. (c) True Beam 6 MV 20 × 20 field Profiles with dip at central axis with FF of Large Field 40 × 40 cm^2^. (d) True Beam 6 MV 40 × 40 field FFF and FF are almost comparable (b) in terms of flatness and dose rate.

Comparison of radiation profiles for smaller FF and larger FF with respect to filter‐free beam is shown in Figure 5. The left side of the figure shows a profile with FFF, the top curve on the right side. Figure 5 shows the flatness achieved by SF, which is flatter till the edges compared to the LF flattener. The resultant SF beam and LF beam show that flatness and symmetry are as per clinical requirements and as per stipulations by regulatory requirements (± 3% for flatness and ± 2% for symmetry for both SF and LF), which were earlier reported^12^ (Figure 5). Comparison of central axis %DD at selected depth for reference fields, for the small filter, and the large filter (Table 3), shows only a large field effect and not much of energy variations. In Figure 6a, the comparison of profiles for Siddharth II FFF and SF with normalization of 100% for 6 MV (FFF), for a 16 × 16 cm^2^ field size is shown. In Figure 6b, the effect of LF for Siddharth II for 30 × 30 cm^2^ field size is shown. For comparison, 6 MV True Beam profiles are represented in Figure 6c,d. Figure 6c compares the variation of beam profiles showing effective flatness at various depths for 20 × 20 cm^2^. Required flatness specification is achieved at 10 cm depth for 20 × 20 cm^2^. Flatness with horns on both sides for flatness curves, at depths < 10 cm upto 1.5 cm (d_max_); less flatness, rounded type curves > 10 cm depths, till 30 cm depths. A careful observation comparing Figure 6b of Siddharth II beam with LF for the 30 × 30 field, and Figure 6d of True Beam of 40 × 40 field exhibits similar pattern in terms of flattening, and also attenuation characteristics.

Comparison of flatness with SF and LF for 10 × 10 cm^2^ in Figure 5b brings out that SF gives better flatness for small fields, and LF gives flatness with more standard deviations. Comparison of Figure 5b and Figure 6b clearly shows that there is a central dip and horns seen in the flatness profile, which do not appear in the use of SF in Siddharth II. In Table 4, the profiles of TrueBeam Linac are analysed to show the Field size effect at various energies. It is clearly seen that single FF in TrueBeam linac gives higher variations in flatness for small field 5 cm × 5cm, for all the photon energies.

**TABLE 4:** Flattening effect of single FF in small and large fields (TrueBeam STX).

			Flatness (%)		Symmetry (%)	
No.	Energy x‐rays	Field Size	Inline	Crossline	Inline	Crossline
1	6 MV	5 × 5 10 × 10 30 × 30	104.12^a^ 102.47 102.28	104.20^a^ 102.43 102.03	100.93 100.66 100.70	100.59 100.20 100.35
2	10 MV	5 × 5 10 × 10 30 × 30	105.46^a^ 102.06 101.71	105.21^a^ 102.27 101.68	101.07 100.79 100.67	100.35 100.93 100.61
3	15 MV	5 × 5 10 × 10 30 × 30	105.43^a^ 101.91 102.06	105.43^a^ 101.88 102.06	101.37 100.68 100.52	101.47 100.51 100.25

^a^ Flatness Limits beyond ± 3.0% with single FF in small fields.

### 3.3 Photon beam characterization for FFF and FF beams

The quantified values of Siddharth II the FFF beams are: Unflatness FFF 16 × 16 75%–100%; Unflatness FFF 30 × 30 60%–100%; Inflection points 16 × 16 7.9–7.9 cm; Inflection points 30 × 30 15.0–15.0 cm; Penumbra FFF 16 × 16 7.5 mm; Penumbra FFF 30 × 30 7.5 mm. For True Beam SVC these values are: Unflatness FFF 20 × 20 72%–100%; Inflection 20 × 20 10.0–10.0 cm; Penumbra FFF 20 × 20 7.9 mm. The quality factors Qi (TPR**_20,10_**) for Siddharth II were: For 16 × 16 field 0.66 (FFF), 0.65 (SF), and 0.71(LF); For 30 × 30 field, 0.65 (FFF) and 0.75 (FF). This clearly shows the effect of beam hardening for bigger flatness filter (LF) and field divergence at the central axis.

### 3.4 Increase in dose rate (dosimetric measurements)

In Table 5, the output factors of FFF and FF beams are brought out. Output factors for FFF and FF beams are matching within 1% with the linac of another design (Varian). From factory original measurements on beam transmission through the SF and LF filters are brought out for all field sizes in Table 6. The average measured transmission against open beam is 0.8106 and 0.4470, respectively for SF and LF flatteners. These measured transmission agrees with theoretically calculated transmission values of 0.7904 (2.5%) and 0.4130 (8.2%) (Case A in Table 2). This indicates a 1.8266 times gain in dose rate output of SF against LF (Case A Table 2). Theoretical estimate of DREF 1.9138 agrees well with 1.8266 within 5%. This equation is based on the effective *µ*‐value for 1.20 MeV, for FFF beam, which might contain a good magnitude of softer photons before reaching copper filters. From Figure 6d it could be observed that a single FF of 6 MV in TrueBeam SVC linac, a reduction of radiation output dose‐rate is about 51% (a factor of 0.5100) of the 6 MV bremsstrahlung beam reaching the flatness filter. Table 7 shows the measured values of transmission for flattened beam in 6 MV, of TrueBeam. Measured transmission of 0.4842 for TrueBeam compares well with 0.5100 ratio taken from measured profiles with and without FF. Siddharth II measurement of transmission with LF (0.4470) compares well with Truebeam transmission of 0.4842. This shows that the SF of Siddharth II linac gives a transmission of 81% which is 30% higher than using a single FF by Varian True Beam SVC.

**TABLE 5:** Output factors at FAD 100 cm, depth = 10 cm (output factors based on same integrated monitor units).

Beam	Field size cm × cm	Siddh‐II	Siddh‐II	True beam	Beam	Field size cm × cm	Siddh‐II	Siddh‐II	True beam
		Iconic	Iconic Plus	SVC			Iconic	Iconic plus	SVC
**6**×**(FF)**	2 × 2 4 × 4 6 × 6 10 × 10 16 × 16 20 × 20 30 × 30 6 × 20 20 × 6	0.800 0.878 0.929 1.000 1.076 1.107 1.155 0.983 0.994	0.796 0.869 0.922 1.000 1.076 1.114 1.171 0.977 0.990	0.799 0.864 0.922 1.000 1.066 1.096 1.139 0.974 0.989	**6**×**(FFF)**	2 × 2 4 × 4 6 × 6 10 × 10 16 × 16 20 × 20 30 × 30 6 × 20 20 × 6	0.809 0.884 0.935 1.000 1.062 1.086 1.116 0.988 0.989	0.805 0.878 0.930 1.000 1.061 1.087 1.123 0.980 0.985	0.790 0.876 0.930 1.000 1.054 1.076 1.106 0.977 0.984

**TABLE 6:** Measured dose for same irradiation time in FFF, SF, and LF settings Siddharth II.

No.	Field cmxcm	FFF cGy	SF cGy	LF cGy	Attenuation SF/FFF	Attenuation LF/FFF	DREF SF/LF
1 2 3 4 5 6 7 8 9	2 × 2 4 × 4 6 × 6 10 × 10 16 × 16 20 × 20 30 × 30 20 × 6 6 × 20	485.76 530.79 561.42 600.45 637.68 652.09 670.10 593.25 593.84	389.62 427.70 453.57 488.24 525.34 – – – –	213.58 234.41 248.02 266.98 287.27 295.55 308.36 262.44 265.38	0.8020 0.8060 0.8080 0.8130 0.8240 – – – – Mean **0.8106**	0.4400 0.4420 0.4420 0.4450 0.4500 0.4570 0.4600 0.4420 0.4470 Mean **0.4470**	1.8227 1.8235 1.8280 1.8269 1.8311 Mean **1.8266**

**TABLE 7:** Estimates of dose rates achieved with FFF and FF in 6 MV in TrueBeam SVC Linac.

		Time for 200 MU with FF Min				Time for 200 MU with FFF min				
No.	Field cm × cm	1	2	Mean Dosr. Rdg. pC	MU/min Estimate	1	2	MU/min Estimate	Mean Dosr. Rdg. pC	Ratio MU/min FF/FFF
1 2 3 4 5 6 7 8	2 × 2 4 × 4 6 × 6 10 × 10 16 × 16 20 × 20 30 × 30 40 × 40	0.303 0.303 0.304 0.304 0.304 0.304 0.304 0.304	0.303 0.304 0.304 0.304 0.304 0.304 0.304 0.304	4540.0 5019.0 5343.2 5781.5 6159.2 6332.0 6591.0 6619.2	660.1 660.1 657.9 657.9 657.9 657.9 657.9 657.9	0.147 0.147 0.147 0.147 0.147 0.147 0.147 0.147	0.147 0.147 0.147 0.147 0.147 0.147 0.147 0.147	1360 1360 1360 1360 1360 1360 1360 1360	4410.3 4846.9 5132.8 5503.0 5801.8 5925.1 6091.7 6106.6	0.4854 0.4854 0.4838 0.4838 0.4838 0.4838 0.4838 0.4838 Mean **0.4842**

## 4 DISCUSSION

This paper has not addressed modifications in FFF beam. With FF treatment plans, in smaller field sizes, in IMRT, VMAT, there will be an added advantage of increased dose rate with better flatness because of using thin flatness filter (reducing effective attenuation of the photon beam). In 1970s, the earlier design of the 42 MeV betatron (Model 500 A, Siemens Erlangen, Germany),^13^, ^14^ use of two lead FFs was reported, one for small fields upto 12 × 12 cm (angle of useful field width α = 0.12) and a second one for large fields upto 16.7 × 16.7 cm (angle of useful field width *α* = 0.167).^13^ A dose rate reduction to 40% and 25% was measured in Homogenizing Filter 1 and 2, respectively, with respect to the 0 Filter condition(because they are lead flatness filters).^13^ With these filters, the output dose rates were about 120 cGy/min at d_max_ at depths of about 4.5 cm.^14^ In similar lines, manufacturers of Siddharth II linac have incorporated two FFs for small and large fields, which is first of its kind in linac designs. The tray insert design in betatron was transitional with 0,1 and 2 positions in sequence. In this Siddharth II model, the SF and LF are kept on either side of open beam port position (Figure 4a). PMT has manufactured Siddharth II linac with two FF filters, to gain the advantage of increased dose rate for fields upto 16 × 16 cm field selection. As most of the smaller tumors are treated with smaller radiation fields, this increased dose rate with better flatness is beneficial. Physics and dosimetric aspects and implications are objectively analysed in this report. Overall increased dose rates for clinical purposes upto 16 × 16 field has long‐term impact on the utilization of linac for clinical purposes, in terms of total filament hours. A theoretical model is explained in this report, taking the mean energy of bremsstrahlung at the entry of FF. As Siddharth II design is first of its kind manufactured in India, we are still working on developing Monte Carlo (MC) model for Siddharth II design, in our information technology (IT) group. Therefore, approximation was made using Varian Linac beam data, and compared for Siddharth II data. Table 6 highlights measured values for MU/min with FFF and FF in 6 MV XRays in TrueBeam SVC Varian Linac. A mean reduction in MU/min value is 0.4842, which is of the same order as 0.4470 measured in LF with Siddharth II Indian linac. From the value of attenuation by copper, evaluated thickness of True Beam FF_6MV_ is 1.54 cm is same order as 1.88 cm of LF (Figure 3a). It is also appreciated from Table 3 that as 10 cm % Depth Dose of FFF is less than FF beam, dosimeter readings are about 2%–3% for integrated 200 MU, but doserates are different by 50%.

It is highlighted from Figure 5b and Figure 6b that the horse saddle type of flatness is not seen in Siddharth II by using a smaller flatness filter, which suppresses less intensity with respect to peripheral part of the circular FF. This will have good effect in achieving more homogeneity in dose distribution. Therefore, in addition to increased dose rates, better flatness is achieved for small fields, more than the effect of large flattener (providing more standard deviations in flatness in small fields). Hence in 6 MV beam, it is expected that the use of SF, will have more efficiency in transmission.

Beam flatness to reduce horns was appreciated in long FSD magna fields total body irradiation (TBI) to achieve uniform flatness, in an earlier report.^15^ In this work, to reduce the effect of edge horns of a 6 MV in extended distances, the beam is further flattened by using polyvinyl chloride (PVC) plates (*ρ* = 1.34 g/cm^3^, 1.5 mm small plate pieces) insertion as a secondary filter in the shadow tray of Clinac 600 CD linear accelerator. By this special FF as a supplement, a beam flatness of 100.16 ± 0.44 was achieved in magna field(200 cm width), compared to open beam flatness of 101.6% ± 1.5% of built‐in FF. By achieving this flatness, in 3 patients a total body dose uniformity of 2.04 ± 4.4%, 1.96 ± 0.8% and 2.01 ± 0.02 Gy against a prescribed dose of 2 Gy.^16^ The TBI example with 40 × 40 opened magna field at 4 meters distance^15^, ^16^ does not have direct relevance to present situation Siddharth II, for two reasons. (1) In O Ring design linacs, extended Focal Skin Distances (telescopic diverged beam) cannot be achieved. (2) Because of isocentre distance restrictions and collimator geometry, maximum field size of 30 × 30 cm only is available in Siddharth II linac specifications. Fine adjustments in flatness definitely improved dose uniformity, suppressing central flatness variations.^15^, ^16^ This example is cited to show that the small flatness filter (SF) in Siddharth II, with really improved flatness in the central part of small fields, will improve to provide better dose volume histogram(DVH).

Dose rate variations in a plane have effects on entrance dose pattern, depth of dose maximum, and central axis depth doses, as having changes in quality and transmissions due to solid angle effects. With the above discussions, it is understood that flatness is crucial in clinical radiotherapy applications, and this is realized in specifications of Siddharth II which is not available in comparable linacs from other manufacturers' designs.

## 5 CONCLUSION

The efficacy of the introduction of an additional flatness filter for smaller radiation fields till 16 × 16 cm^2^ is brought out in this report, which provided better flatness as well as increased dose rate. Most of the curative radical treatments employ smaller field sizes, and therefore arrived higher dose rates will reduce the time of irradiation, helping to meet immobilization requirements. From the long usage point of view of the linac, overall reduction in cathode hours will extend life of the related electronics. There are no reports available in the literature regarding flatness filters, and therefore, this work is first of its kind, highlighting the energy of photons involved, their penetration details, and related measurements.

## AUTHOR CONTRIBUTIONS

All authors have contributed in the design, preparation, discussion of the entire script material experiments and data collection.

## CONFLICT OF INTEREST STATEMENT

The authors declare no conflicts of interest.

## ETHICS STATEMENT

There are no ethical considerations in this scientific article.

## Data Availability

All related data are preserved in the factory and with first author.
